# Bidirectional Associations Between Seborrheic Dermatitis and Epithelial Barrier Diseases: A Retrospective Cohort Study

**DOI:** 10.1111/all.70112

**Published:** 2025-10-27

**Authors:** Sabrina Meng, Ronald Berna, Junko Takeshita, Ole Hoffstad, Daniel Shin, Nandita Mitra, David J. Margolis

**Affiliations:** ^1^ Perelman School of Medicine University of Pennsylvania Philadelphia Pennsylvania USA; ^2^ Department of Dermatology Perelman School of Medicine at the University of Pennsylvania Philadelphia Pennsylvania USA; ^3^ Department of Biostatistics and Epidemiology Perelman School of Medicine at the University of Pennsylvania Philadelphia Pennsylvania USA

**Keywords:** atopic disease, epithelial barrier theory, inflammatory diseases, seborrheic dermatitis

## Abstract

**Background:**

Seborrheic dermatitis (SD) is a common skin disease characterized by epithelial barrier breakdown. The epithelial barrier theory (EBT) implicates epithelial barrier disruption in the development of epithelial barrier diseases (EBDs). We hypothesized that SD is associated with an increased risk of EBDs and investigated the bidirectional association between SD and EBDs.

**Methods:**

Retrospective cohort study of 5,083,689 patients using a large US administrative claims database from January 1, 2016, to June 30, 2022. The mean (standard deviation) follow‐up was 3.25 (1.75) years with a total follow‐up of over 16 million person‐years. The outcomes were a diagnosis of (i) SD after an EBD and (ii) EBD after SD, analyzed using a multivariable Cox proportional hazards model (hazard ratio [95% confidence interval]).

**Results:**

The risk of SD was increased after EBD diagnosis for multiple diseases, including atopic dermatitis [2.46 (2.40, 2.53)], alopecia areata [3.47 (3.24, 3.71)], contact dermatitis [1.92 (1.88, 1.96)], psoriasis [2.62 (2.54, 2.69)], rosacea [2.84 (2.78, 2.90)], hidradenitis suppurativa [1.79 (1.63, 1.97)], rhinosinusitis [1.34 (1.32, 1.35)], food allergy [1.47 (1.42, 1.54)], celiac disease [1.55 (1.43, 1.68)], ocular allergy [1.55 (1.49, 1.61)], and dry eye [1.54 (1.52, 1.56)]. The risk of EBD after SD diagnosis followed similar trends, with the largest effect estimates being psoriasis [3.52 (3.42, 3.61)], rosacea [2.85 (2.79, 2.92)], and alopecia areata [2.81 (2.61, 3.03)].

**Conclusions:**

Our results support the EBT as a shared driver of EBD pathogenesis at not only local (e.g., skin) barriers but also at non‐local sites, including the respiratory, gastrointestinal, and ocular epithelial barriers.

## Graphical abstract

This study explores the bidirectional associations between SD and EBDs in a cohort of 5,083,689 individuals in a US administrative claims database. Positive bidirectional associations were found between SD and many EBDs of the skin, respiratory, gastrointestinal, and ocular surfaces. Our results support the EBT as a shared driver of EBD pathogenesis at both local and non‐local epithelial barriers. EBD, epithelial barrier disease; EBT, epithelial barrier theory; GERD, gastroesophageal reflux disease; SD, seborrheic dermatitis; US, United States.

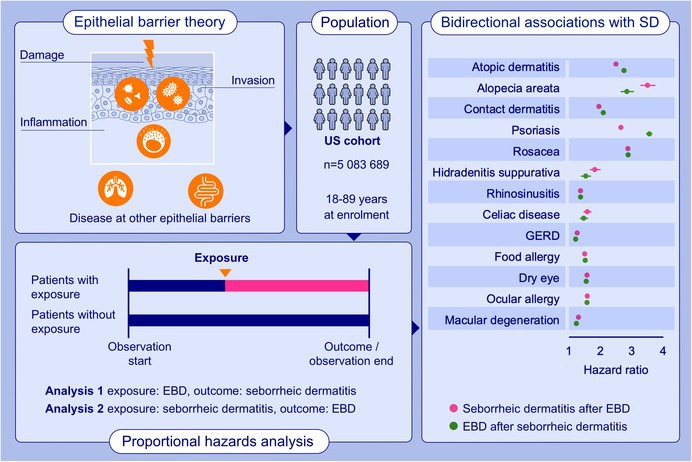

## 1 Introduction

Seborrheic dermatitis (SD) is a common dermatologic condition that presents with erythema with overlying greasy scale primarily located on the face, scalp, and other areas with high sebaceous gland activity [1]. SD first occurs in infancy, adolescence, or adulthood, leading to chronic inflammatory skin disease in approximately 5% of the population worldwide [2, 3]. SD is characterized by skin barrier disruption, which is supported by electron microscopy studies showing a disorganized stratum corneum, as well as functional studies demonstrating an increase in trans‐epidermal water loss [4, 5]. Further evidence for skin barrier disruption in SD includes symptoms of itching and flaking, which are hypothesized to arise from factors such as colonization by *Malassezia* yeast, changes in skin lipid composition, and immunologic response against pathologic free fatty acids and lipid peroxides [6, 7, 8, 9, 10].

Epithelial barrier disruption has been implicated in the development of many epithelial barrier diseases (EBDs) of the skin, respiratory tract, gastrointestinal tract, and ocular surface [11]. The epithelial barrier theory (EBT) proposes that EBDs arise from epithelial barrier breakdown, which results in tissue exposure to pathogens and environmental allergens. These exposures lead to pathological events that include pathogen and allergen migration to subepithelial areas, subsequent immune response, and impaired epithelial barrier healing, which leads to an inflammatory state that is believed to be a contributing factor to the development of chronic inflammatory conditions [11, 12, 13, 14].

Thus, we hypothesize that SD‐associated epithelial barrier breakdown may induce a chronic inflammatory state that increases the risk of other EBDs, many of which are immunologically mediated [11]. Moreover, given that many EBDs result in epithelial barrier dysfunction and that SD is also an inflammatory disease, we believe that chronic inflammatory states induced by EBDs may increase the likelihood of developing SD as well [15]. Some studies have investigated the association between SD and certain dermatologic diseases and ocular diseases, as well as Parkinson's disease [16, 17, 18]. However, the relationship between SD and many EBDs remains unclear, and the directional association is even less well understood. For example, it is unknown whether the association between SD and EBDs primarily results from initial SD‐related epithelial barrier disruption, initial EBD‐related epithelial barrier disruption, or simultaneous disruption. Elucidating the directional association between SD and EBDs can also help us better understand the sequence of inflammatory events that are implicated in the EBT model of disease [11, 19]. In this study, we aim to examine the bidirectional association between SD and dermatologic, gastrointestinal, respiratory, and ocular EBDs using a large US administrative claims database, thus filling a gap in the literature.

## 2 Methods

We examined a cohort of 5,083,689 patients from Optum's de‐identified Clinformatics Data Mart Database (Optum CDM) limited to patients of at least 18 years of age and with at least three visits on unique days to a medical professional during their first 180 days of enrollment. This cohort was created to capture a population of high healthcare utilizers who are likely to receive a timely diagnosis after disease onset. Patients with diagnoses of SD or any EBDs in the initial 180‐day observation period were excluded to ensure that no exposures or outcomes of interest were observed before the analysis period. Patients with less than 1 year of continuous follow‐up between January 1, 2016, and June 30, 2022, representing the time frame of claims data available using ICD‐10 coding, after the 180‐day observation period were excluded. We excluded individuals with missing data for any of the demographic covariates: age, sex, race (White vs. non‐White), and division (i.e., billing region). Additional details on methods and cohort construction can be found in Supporting Methods (Figure S1).

Our selection of EBDs was derived from those published by Sun et al. [11]. The prevalence of each disease was determined by ICD‐10 coding, and codes were verified against codes used in published literature whenever possible [20, 21, 22, 23, 24, 25, 26, 27, 28, 29, 30, 31, 32, 33, 34, 35, 36, 37, 38]. Information on the ICD‐10 codes used to define each disease can be found in Table S1. Unless otherwise specified, we defined a patient as having SD or an EBD if they had at least one ICD‐10 diagnosis code for the corresponding disease logged at a medical encounter.

**FIGURE 1:**
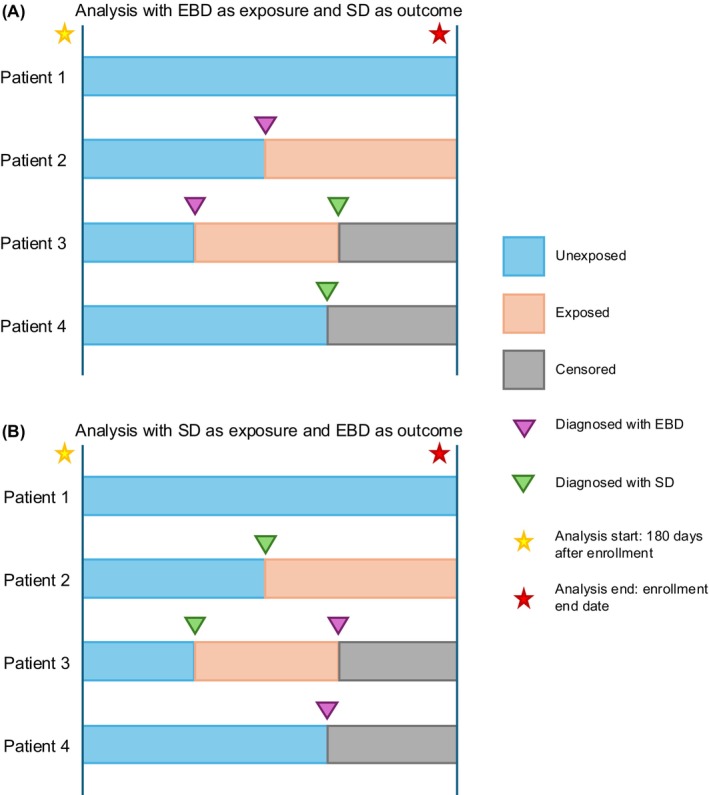
Sample timelines for patient 1 (no exposure or outcome), patient 2 (exposure only), patient 3 (exposure and outcome), and patient 4 (outcome only) for (A) the analysis with EBD as exposure and SD as outcome and (B) the analysis with SD as exposure and EBD as outcome. All patients were censored at the end of the analysis period if they were not already censored.

The outcome for the first analysis was (i) the diagnosis of SD, with EBD as the exposure, and the outcome for the second analysis was (ii) the diagnosis of EBD, with SD as the exposure. A Cox proportional hazards model was used to examine the association between SD and each EBD via the hazard ratio (HR) and 95% confidence interval (CI). Unless otherwise specified, the association between SD and each EBD was investigated independently of other EBDs. Analyses were completed with disease exposure as a time‐varying variable. Patients were censored after the observation of the outcome or at the end of their enrollment period, whichever was earlier. A sample of various patient exposure and censorship timelines can be found in Figure 1. Given the large size of the cohort, we emphasized the clinical significance of the HR instead of considering only the magnitude of the *p*‐values. For this reason, any effect estimate, including its corresponding 95% CI, that was not ≥ 1.15 or ≤ 0.85 was not considered to be clinically significant a priori.

Multivariable analysis in the survival models included age at enrollment, sex, race (White vs. non‐White), and division (i.e., billing region) at enrollment. We performed sensitivity analyses with (i) adjustment for a concurrent diagnosis of atopic dermatitis (AD) or psoriasis and (ii) exclusion of individuals with AD or psoriasis to evaluate possible bias from misdiagnosis of SD as AD or psoriasis. We additionally performed (iii) an analysis in which the diagnosis of SD and EBDs required an ICD‐10 code to be logged at two separate encounters instead of one. The purpose of this analysis was to evaluate the impact of a stronger requirement for diagnosis on the observed associations and to mitigate bias from possible misdiagnosis. We performed (iv) an analysis in which the cohort included patients who had at least one encounter within their 180‐day observation period instead of three. The purpose of this analysis was to observe the impact of differential healthcare utilization on effect sizes. Finally, we performed analyses evaluating the impact of (v) comorbid type 2 diabetes and obesity and (vi) smoking and alcohol use disorder.

All analyses were conducted using Stata MP version 18. This study was exempt per the University of Pennsylvania Institutional Review Board.

## 3 Results

The characteristics of the cohort are summarized in Table 1. Characteristics of additional sub‐cohorts used in secondary analyses can be found in Tables S2–S4. In the cohort of 5,083,689 individuals, 181,478 individuals had at least one diagnosis of SD during their enrollment period, for a prevalence of 3.57%. The mean (standard deviation) follow‐up time after the 180‐day observation period was 3.25 (1.75) years with a total follow‐up of over 16 million person‐years. Patients diagnosed with SD were more frequently male (46.20%) and White (80.45%) and older (median = 64.55 years) when compared to the full cohort (40.98%, 73.44%, and 57.54 years, respectively). The hazard ratio (HR) and 95% confidence interval (CI) for the risk of SD diagnosis after EBD diagnosis and the risk of EBD diagnosis after SD diagnosis using a multivariable Cox proportional hazards model are shown in Table 2 and Table 3, respectively, along with disease prevalences. The effect estimates for secondary analyses can be found in Tables S5–S8.

**TABLE 1:** Summary of characteristics for the full cohort, patients with a SD diagnosis, and patients without a SD diagnosis.

	Full cohort *N* = 5,083,689	SD diagnosis *N* = 181,478	No SD diagnosis *N* = 4,902,211
Age at enrollment (years), median (IQR)	57.54 (40.53, 67.64)	64.55 (44.53, 70.55)	57.46 (40.53, 67.55)
Sex, count (% total)			
Female	3,000,186 (59.02%)	97,642 (53.80%)	2,902,544 (59.21%)
Male	2,083,503 (40.98%)	83,836 (46.20%)	1,999,667 (40.79%)
Race, count (% total)			
White	3,733,651 (73.44%)	145,992 (80.45%)	3,587,659 (73.18%)
Non‐White	1,350,038 (26.56%)	35,486 (19.55%)	1,314,552 (26.82%)
Division at time of enrollment, count (% total)			
East North Central (IL, IN, MI, OH, WI)	735,732 (14.47%)	22,214 (12.24%)	713,518 (14.56%)
East South Central (AL, KY, MS, TN)	222,478 (4.38%)	7278 (4.01%)	215,200 (4.39%)
Middle Atlantic (NJ, NY, PA)	417,151 (8.21%)	15,618 (8.61%)	401,533 (8.19%)
Mountain (AZ, CO, ID, MT, NV, NM, UT, WY)	486,606 (9.57%)	16,286 (8.97%)	470,320 (9.59%)
New England (CT, ME, MA, NH, RI, VT)	209,672 (4.12%)	7165 (3.95%)	202,507 (4.13%)
Pacific (AK, CA, HI, OR, WA)	606,121 (11.92%)	23,100 (12.73%)	583,021 (11.89%)
South Atlantic (DE, DC, FL, GA, MD, NC, SC, VA, WV)	1,205,337 (23.71%)	54,296 (29.92%)	1,151,041 (23.48%)
West North Central (IA, KS, MN, MO, NE, ND, SD)	476,609 (9.38%)	13,325 (7.34%)	463,284 (9.45%)
West South Central (AR, LA, OK, TX)	723,983 (14.24%)	22,196 (12.23%)	701,787 (14.32%)

**TABLE 2:** EBD prevalences and the hazard ratio (HR) and 95% confidence interval (CI) for the risk of SD diagnosis after EBD diagnosis using a multivariable Cox proportional hazards model, both unadjusted and fully adjusted for age, sex, race, and division.

Disease	Unadjusted HR (95% CI)	Unadjusted, *p*‐value^a^	Adjusted HR (95% CI)	Adjusted, *p*‐value^a^	Prevalence (%)
**Skin**					
Atopic dermatitis (AD)	2.48 (2.41, 2.54)	< 0.001	2.46 (2.40, 2.53)	< 0.001	2.55
Alopecia areata (AA)	3.20 (2.99, 3.43)	< 0.001	3.47 (3.24, 3.71)	< 0.001	0.28
Contact dermatitis	1.94 (1.91, 1.98)	< 0.001	1.92 (1.88, 1.96)	< 0.001	5.44
Psoriasis	2.72 (2.64, 2.80)	< 0.001	2.62 (2.54, 2.69)	< 0.001	1.78
Rosacea	2.90 (2.83, 2.96)	< 0.001	2.84 (2.78, 2.90)	< 0.001	2.88
Hidradenitis suppurativa	1.58 (1.44, 1.74)	< 0.001	1.79 (1.63, 1.97)	< 0.001	0.28
**Respiratory**					
Asthma	1.13 (1.10, 1.15)	< 0.001	1.17 (1.14, 1.19)	< 0.001	7.38
Rhinosinusitis	1.32 (1.30, 1.34)	< 0.001	1.34 (1.32, 1.35)	< 0.001	20.03
Chronic obstructive pulmonary disease (COPD)	1.00 (0.98, 1.02)	> 0.99	0.92 (0.90, 0.94)	< 0.001	6.76
Sarcoidosis	1.26 (1.11, 1.42)	0.005	1.25 (1.11, 1.41)	0.008	0.17
Pulmonary hypertension	1.04 (1.00, 1.08)	0.65	0.97 (0.93, 1.00)	> 0.99	3.03
**Gastrointestinal**					
Eosinophilic esophagitis	1.28 (1.14, 1.44)	< 0.001	1.27 (1.13, 1.43)	< 0.001	0.21
Gastroesophageal reflux disease (GERD)	1.27 (1.25, 1.28)	< 0.001	1.23 (1.21, 1.24)	< 0.001	22.75
Food allergy	1.43 (1.37, 1.49)	< 0.001	1.47 (1.42, 1.54)	< 0.001	1.65
Inflammatory bowel disease (IBD)	1.26 (1.19, 1.32)	< 0.001	1.22 (1.16, 1.28)	< 0.001	1.08
Celiac disease	1.51 (1.39, 1.64)	< 0.001	1.55 (1.43, 1.68)	< 0.001	0.35
Diverticulosis	1.29 (1.27, 1.31)	< 0.001	1.21 (1.19, 1.22)	< 0.001	14.71
**Ocular**					
Ocular allergy	1.49 (1.43, 1.55)	< 0.001	1.55 (1.49, 1.61)	< 0.001	1.43
Macular degeneration	1.40 (1.37, 1.43)	< 0.001	1.27 (1.24, 1.30)	< 0.001	5.18
Dry eye	1.58 (1.55, 1.60)	< 0.001	1.54 (1.52, 1.56)	< 0.001	13.47
Glaucoma	1.20 (1.18, 1.22)	< 0.001	1.16 (1.14, 1.18)	< 0.001	8.40
Uveitis	1.24 (1.17, 1.31)	< 0.001	1.23 (1.16, 1.31)	< 0.001	0.79

^a^ All *p* values are reported with Bonferroni correction for 22 hypotheses.

**TABLE 3:** EBD prevalences and the hazard ratio (HR) and 95% confidence interval (CI) for the risk of EBD diagnosis after SD diagnosis using a multivariable Cox proportional hazards model, both unadjusted and fully adjusted for age, sex, race, and division.

Disease	Unadjusted HR (95% CI)	Unadjusted, *p*‐value^a^	Adjusted HR (95% CI)	Adjusted, *p*‐value^a^	Prevalence (%)
**Skin**					
Atopic dermatitis (AD)	2.72 (2.66, 2.78)	< 0.001	2.72 (2.66, 2.78)	< 0.001	2.55
Alopecia areata (AA)	2.63 (2.44, 2.83)	< 0.001	2.81 (2.61, 3.03)	< 0.001	0.28
Contact dermatitis	2.08 (2.04, 2.12)	< 0.001	2.06 (2.02, 2.10)	< 0.001	5.44
Psoriasis	3.65 (3.56, 3.76)	< 0.001	3.52 (3.42, 3.61)	< 0.001	1.78
Rosacea	2.87 (2.80, 2.94)	< 0.001	2.85 (2.79, 2.92)	< 0.001	2.88
Hidradenitis suppurativa	1.36 (1.23, 1.51)	< 0.001	1.50 (1.35, 1.67)	< 0.001	0.28
**Respiratory**					
Asthma	1.14 (1.12, 1.17)	< 0.001	1.18 (1.16, 1.21)	< 0.001	7.38
Rhinosinusitis	1.32 (1.31, 1.34)	< 0.001	1.34 (1.32, 1.36)	< 0.001	20.03
Chronic obstructive pulmonary disease (COPD)	1.06 (1.04, 1.09)	< 0.001	0.93 (0.91, 0.95)	< 0.001	6.76
Sarcoidosis	1.37 (1.19, 1.56)	< 0.001	1.34 (1.17, 1.53)	< 0.001	0.17
Pulmonary hypertension	1.07 (1.04, 1.11)	< 0.001	0.95 (0.92, 0.98)	< 0.03	3.03
**Gastrointestinal**					
Eosinophilic esophagitis	1.37 (1.22, 1.54)	< 0.001	1.38 (1.23, 1.54)	< 0.001	0.21
Gastroesophageal reflux disease (GERD)	1.23 (1.21, 1.24)	< 0.001	1.18 (1.17, 1.20)	< 0.001	22.75
Food allergy	1.44 (1.39, 1.50)	< 0.001	1.49 (1.43, 1.55)	< 0.001	1.65
Inflammatory bowel disease (IBD)	1.26 (1.19, 1.32)	< 0.001	1.21 (1.14, 1.27)	< 0.001	1.08
Celiac disease	1.40 (1.27, 1.54)	< 0.001	1.44 (1.31, 1.58)	< 0.001	0.35
Diverticulosis	1.28 (1.26, 1.30)	< 0.001	1.17 (1.15, 1.19)	< 0.001	14.71
**Ocular**					
Ocular allergy	1.47 (1.41, 1.53)	< 0.001	1.54 (1.48, 1.60)	< 0.001	1.43
Macular degeneration	1.37 (1.34, 1.40)	< 0.001	1.21 (1.18, 1.24)	< 0.001	5.18
Dry eye	1.54 (1.52, 1.56)	< 0.001	1.52 (1.49, 1.54)	< 0.001	13.47
Glaucoma	1.17 (1.14, 1.19)	< 0.001	1.12 (1.10, 1.15)	< 0.001	8.40
Uveitis	1.21 (1.14, 1.29)	< 0.001	1.20 (1.13, 1.27)	< 0.001	0.79

^a^ All *p* values are reported with Bonferroni correction for 22 hypotheses.

Using fully adjusted analyses, the risk of SD diagnosis was increased after EBD diagnosis for all dermatologic conditions [HR (95% CI)]: AD [2.46 (2.40, 2.53)], alopecia areata (AA) [3.47 (3.24, 3.71)], contact dermatitis [1.92 (1.88, 1.96)], psoriasis [2.62 (2.54, 2.69)], rosacea [2.84 (2.78, 2.90)], and hidradenitis suppurativa [1.79 (1.63, 1.97)]. The risk of SD was also increased after rhinosinusitis [1.34 (1.32, 1.35)], gastroesophageal reflux disease (GERD) [1.23 (1.21, 1.24)], food allergy [1.47 (1.42, 1.54)], inflammatory bowel disease (IBD) [1.22 (1.16, 1.28)], celiac disease [1.55 (1.43, 1.68)], diverticulosis [1.21 (1.19, 1.22)], ocular allergy [1.55 (1.49, 1.61)], macular degeneration [1.27 (1.24, 1.30)], dry eye [1.54 (1.52, 1.56)], and uveitis [1.23 (1.16, 1.31)].

Similarly, using fully adjusted analyses, the risk of EBD diagnosis was increased after SD diagnosis for all dermatologic conditions: AD [2.72 (2.66, 2.78)], AA [2.81 (2.61, 3.03)], contact dermatitis [2.06 (2.02, 2.10)], psoriasis [3.52 (3.42, 3.61)], rosacea [2.85 (2.79, 2.92)], and hidradenitis suppurativa [1.50 (1.35, 1.67)]. After SD diagnosis, there was also an increased risk of asthma [1.18 (1.16, 1.21)], rhinosinusitis [1.34 (1.32, 1.36)], sarcoidosis [1.34 (1.17, 1.53)], eosinophilic esophagitis [1.38 (1.23, 1.54)], GERD [1.18 (1.17, 1.20)], food allergy [1.49 (1.43, 1.55)], celiac disease [1.44 (1.31, 1.58)], ocular allergy [1.54 (1.48, 1.60)], macular degeneration [1.21 (1.18, 1.24)], and dry eye [1.52 (1.49, 1.54)].

We found directional differences in the association between SD and select dermatologic diseases. The risk of psoriasis [3.52 (3.42, 3.61)], AD [2.72 (2.66, 2.78)], and contact dermatitis [2.06 (2.02, 2.10)] after SD was elevated compared to the risk of SD after psoriasis [2.62 (2.54, 2.69)], AD [2.46 (2.40, 2.53)], and contact dermatitis [1.92 (1.88, 1.96)]. In contrast, the risk of SD after AA [3.47 (3.24, 3.71)] was elevated compared to the risk of AA after SD [2.81 (2.61, 3.03)].

## 4 Discussion

The pathogenesis of SD is a complex process that results in skin barrier disruption, immune response, and chronic inflammation. The EBT has proposed that many EBDs of the skin, respiratory tract, gastrointestinal tract, and ocular surface arise from epithelial barrier disruption in a similar manner [11]. To characterize the relationship between SD and EBDs and to understand the possibility of a shared pathogenetic mechanism between SD and EBDs, we evaluated the bidirectional association between SD and EBD diagnosis using a multivariable Cox proportional hazards model.

We found that the risk of developing both SD after EBDs and EBDs after SD was elevated for dermatologic conditions. Positive associations between SD and AD, psoriasis, rosacea, and hidradenitis suppurativa have previously been reported [2, 16, 39, 40]. However, associations were variable, with some studies finding associations between SD and AD in children and infants but not adults, for example [2, 16, 41]. The association of SD with AA is also variable and has not been widely studied [42, 43]. Thus, our findings both support existing associations between SD and dermatologic EBDs and elucidate novel relationships for EBDs that lack firmly established associations with SD. Moreover, differences in directional associations between SD and psoriasis, AD, and contact dermatitis suggest that SD is more likely to be diagnosed before these diseases or that these diseases are commonly first misdiagnosed as SD. While a mechanism linking AA and seborrheic dermatitis has not been reported, the difference in directional association between SD and AA indicates that AA is more likely to be diagnosed before SD or that AA exposes additional skin surface area, thereby facilitating SD diagnosis. However, other explanations such as differences in diagnostic frequency among EBDs cannot be excluded.

The positive bidirectional associations between SD and diverse dermatologic conditions lend support to the EBT model, suggesting that epithelial barrier disruption contributes to the pathogenesis of EBDs with diverse inflammatory mechanisms. Additionally, studies have found shared inflammatory markers between SD and psoriasis (IL‐1, IL‐17, and TNF); AD (IL‐17, IL‐1, and IL‐4); contact dermatitis (IL‐1, TNF, and IL‐8); rosacea (IL‐1 and TNF); hidradenitis suppurativa (IL‐1 and IL‐17); and AA (IL‐1 and TNF) [44, 45, 46, 47, 48, 49]. Thus, we hypothesize that inflammatory markers such as IL‐1, IL‐17, and TNF may be important in the pathogenesis of EBDs and warrant future study.

We also discovered bidirectional associations between SD and multiple respiratory, gastrointestinal, and ocular EBDs. While many of these associations have not been previously studied, our findings corroborate associations between SD and dry eye [17]. The mechanism behind associations between SD and non‐dermatologic EBDs may be explained by the skin‐gut, skin‐lung, and skin‐eye axes. For example, the skin‐gut axis theorizes that damage to the skin leads to changes in systemic host defenses and alterations in the gut microbiome, and vice versa [50, 51, 52]. While the skin‐lung axis has not been widely studied, theories about the lung microbiome and gut‐lung axis have emerged, in which immune dysregulation at the gut mucosa can lead to systemic changes that influence respiratory health [53, 54, 55]. Given the evidence for both the skin‐gut axis and gut‐lung axis, it is plausible that disruptions of the skin can also influence respiratory health via a skin‐lung axis. Similarly, the role of the gut microbiome in immune system function, ocular barrier homeostasis, and ocular diseases has led to the proposal of a gut‐eye axis [56, 57, 58]. The combination of the skin‐gut and gut‐eye axes implies that there may exist a skin‐eye axis. Further investigation is needed to ascertain the mechanisms governing the associations between SD and non‐dermatologic EBDs.

Notably, some diseases were not strongly associated with seborrheic dermatitis, including chronic obstructive pulmonary disease (COPD), pulmonary hypertension (PH), and glaucoma. In COPD and PH, proposed mechanisms of disease include exposure to environmental insults and increased respiratory epithelium permeability [11, 59]. However, our results indicate that the inflammatory processes in these diseases may be orthogonal to those seen in conditions like SD or that other risk factors (e.g., genetics) contribute more significantly to disease pathogenesis [60, 61]. Similarly, glaucoma has established genetic etiologies [62, 63]. Importantly, while epithelial barrier dysfunction can be an important risk factor for epithelial barrier diseases, each disease also has its own specific mechanisms, risk factors, and genetic predispositions that contribute to pathogenesis.

Our results were robust to many sensitivity analyses evaluating the impact of possible disease misclassification, comorbid diseases, and environmental factors. Our sensitivity analyses (i) adjusting for AD and psoriasis, (ii) removing patients with AD and psoriasis, and (iii) using two diagnosis codes for each disease yielded results similar to those in the primary analysis (Tables S5 and S6), which indicate that misclassification is not likely to have a large impact on results. Additionally, most associations between SD and EBDs strengthened when a stronger criterion of two diagnosis codes was used to define disease (Tables S5 and S6), further supporting the link between SD and EBDs. Moreover, adjusting for comorbid conditions such as type 2 diabetes and obesity, and environmental exposures such as smoking and alcohol use, did not have a meaningful impact on results (Tables S7 and S8). These findings indicate that associations between SD and EBDs are unlikely to be explained by non‐biological factors and support the theory of shared pathogenetic mechanisms among EBDs.

Our study faces several limitations. Because the Optum CDM is not a birth cohort, it is possible that patients have previously diagnosed diseases that are not recorded in the dataset, thus affecting risk estimates. We mitigated this bias by introducing an initial 180‐day observation period and removing patients who have diagnoses recorded for SD and EBDs before the analysis period. Our use of an administrative database excludes uninsured populations and healthy individuals who do not regularly see a medical professional, and gaps in insurance coverage lead to termination of the follow‐up period, which can alter the magnitude of effect sizes. Moreover, claims data is collected only when an individual interacts with the healthcare system, so diagnoses can be missed or delayed. We mitigated this bias by building a cohort of high healthcare utilizers who are likely to have a timely disease diagnosis; we also show that the results of a high healthcare utilization cohort do not differ substantially when compared to a lower healthcare utilization cohort. Our analysis lacked validated codes for many diseases, did not assess the severity and chronicity of SD, and used ICD‐10 codes rather than physician‐confirmed assessments for diagnosis, which may increase the risk of misclassification bias. We mitigated the possibility of misclassification bias with a sensitivity analysis defining each disease using two ICD‐10 codes. Finally, because SD was found to be strongly associated with dermatological EBDs, the possibility of comorbid dermatological EBDs contributing to the associations between SD and other EBDs cannot be excluded.

Our study possesses several strengths. First, we used a large cohort of over five million individuals representing a diverse range of age, sex, race, and geographic locations across the US. Additionally, we followed patients longitudinally in the Optum CDM from January 1, 2016, to June 30, 2022, resulting in up to 5 years of longitudinal follow‐up excluding the initial 180‐day observation period. Finally, our study not only investigates associations between SD and dermatologic conditions, but also expands the scope to gastrointestinal, respiratory, and ocular EBDs, thus addressing a gap in the literature.

In conclusion, SD is a common disease of the skin that leads to epithelial barrier breakdown. While its pathophysiology is not yet fully understood, it is associated with EBDs of the skin, gastrointestinal, respiratory, and ocular barriers, lending support to the EBT. Additionally, our studies demonstrated differences in bidirectional associations between SD and certain EBDs, indicating possible temporality in disease progression. We also identify EBDs that are not strongly associated with SD, which suggests that, in these diseases, epithelial barrier disruption may result in inflammatory processes orthogonal to those in SD or that other risk factors play an important role in pathogenesis. Future research may include longitudinal prospective cohort studies to ascertain the association between SD and EBDs. Mechanistic studies investigating immunological profiles in individuals with and without disease may also be performed to confirm the inflammatory mechanisms involved in SD and EBD pathogenesis.

## Author Contributions

Conceptualization: S.M., D.J.M., R.B., J.T., O.H., D.S., N.M. Data Curation: S.M., O.H., D.S., D.J.M., R.B. Investigation: S.M., R.B., D.J.M. Methodology: S.M., R.B., D.J.M., J.T., N.M. Writing – Original Draft: S.M. Writing – Revision and Editing: S.M., D.J.M., R.B., N.M., J.T., D.S., O.H.

## Ethics Statement

Reviewed and exempt per the University of Pennsylvania Institutional Review Board.

## Conflicts of Interest

J.T. receives research grants (paid to the Trustees of the University of Pennsylvania) from Pfizer Inc. and Bristol Myers Squibb and has served as a consultant for Incyte and the National Eczema Association, receiving honoraria. D.J.M. has ongoing or recent consulting relationships with GSK, MSD, INSMED, and Janssen. MSD, INSMED, and Janssen are associated with membership of DSMB. He receives research funding via the Trustees of the University of Pennsylvania from Bausch and the NIH. None of his potential conflicts of interest are associated with the topics discussed in the current study. No other disclosures were reported.

## Supporting information


**Figure S1:** Schematic of analytical cohort construction showing the number of patients at each stage of cohort construction.
**Table S1:** Table of diseases and corresponding ICD‐10 codes.
**Table S2:** Cohort characteristics when defining SD diagnosis using at least two instances of SD‐related ICD‐10 codes.
**Table S3:** Cohort characteristics after removing AD and psoriasis patients.
**Table S4:** Cohort characteristics for an expanded cohort containing patients with at least one encounter during the initial 180‐day observation period.
**Table S5:** The hazard ratio (HR) and 95% confidence interval (CI) of SD diagnosis after EBD diagnosis using a multivariable Cox proportional hazards model for the following secondary analyses: adjusting for AD and psoriasis, removing AD and psoriasis patients, defining SD with at least two instances of diagnosis codes instead of one, and utilizing an expanded cohort of patients with at least one encounter during the 180‐day observation period.
**Table S6:** The hazard ratio (HR) and 95% confidence interval (CI) of EBD diagnosis after SD diagnosis using a multivariable Cox proportional hazards model for the following secondary analyses: adjusting for AD and psoriasis, removing AD and psoriasis patients, defining SD with at least two instances of diagnosis codes instead of one, and utilizing an expanded cohort of patients with at least one encounter during the 180‐day observation period.
**Table S7:** The hazard ratio (HR) and 95% confidence interval (CI) of SD diagnosis after EBD diagnosis using a multivariable Cox proportional hazards model for the following secondary analyses: adjusting for comorbid obesity and type 2 diabetes and additionally adjusting for environmental exposures to smoking and alcohol.
**Table S8:** The hazard ratio (HR) and 95% confidence interval (CI) of EBD diagnosis after SD diagnosis using a multivariable Cox proportional hazards model for the following secondary analyses: adjusting for comorbid obesity and type 2 diabetes and additionally adjusting for environmental exposures to smoking and alcohol.

## Data Availability

The data used for this study were purchased from and their use is regulated by Optum Inc. through agreement with the Leonard Davis Institute of the University of Pennsylvania. The authors cannot share the primary data, but the data are available by purchase from Optum Inc.
