# Supplementary material for: Bidirectional Associations Between Seborrheic Dermatitis and Epithelial Barrier Diseases: A Retrospective Cohort Study
Source: Allergy. 2025 Oct 27;81(3):808–17. doi: 10.1111/all.70112 (PMC12869902; doi:10.1111/all.70112)
Supplement: Supplementary file 1 — Figure S1: Schematic of analytical cohort construction showing the number of patients at each stage of cohort construction. Table S1: Table of diseases and corresponding ICD‐10 codes. Table S2: Cohort characteristics when defining SD diagnosis using at least two instances of SD‐related ICD‐10 codes. Table S3: Cohort characteristics after removing AD and psoriasis patients. Table S4: Cohort characteristics for an expanded cohort containing patients with at least one encounter during the initial 180‐day observation period. Table S5: The hazard ratio (HR) and 95% confidence interval (CI) of SD diagnosis after EBD diagnosis using a multivariable Cox proportional hazards model for the following secondary analyses: adjusting for AD and psoriasis, removing AD and psoriasis patients, defining SD with at least two instances of diagnosis codes instead of one, and utilizing an expanded cohort of patients with at least one encounter during the 180‐day observation period. Table S6: The hazard ratio (HR) and 95% confidence interval (CI) of EBD diagnosis after SD diagnosis using a multivariable Cox proportional hazards model for the following secondary analyses: adjusting for AD and psoriasis, removing AD and psoriasis patients, defining SD with at least two instances of diagnosis codes instead of one, and utilizing an expanded cohort of patients with at least one encounter during the 180‐day observation period. Table S7: The hazard ratio (HR) and 95% confidence interval (CI) of SD diagnosis after EBD diagnosis using a multivariable Cox proportional hazards model for the following secondary analyses: adjusting for comorbid obesity and type 2 diabetes and additionally adjusting for environmental exposures to smoking and alcohol. Table S8: The hazard ratio (HR) and 95% confidence interval (CI) of EBD diagnosis after SD diagnosis using a multivariable Cox proportional hazards model for the following secondary analyses: adjusting for comorbid obesity and type 2 diabetes and additional [file ALL-81-808-s001.docx]

**Supplemental Methods**

**
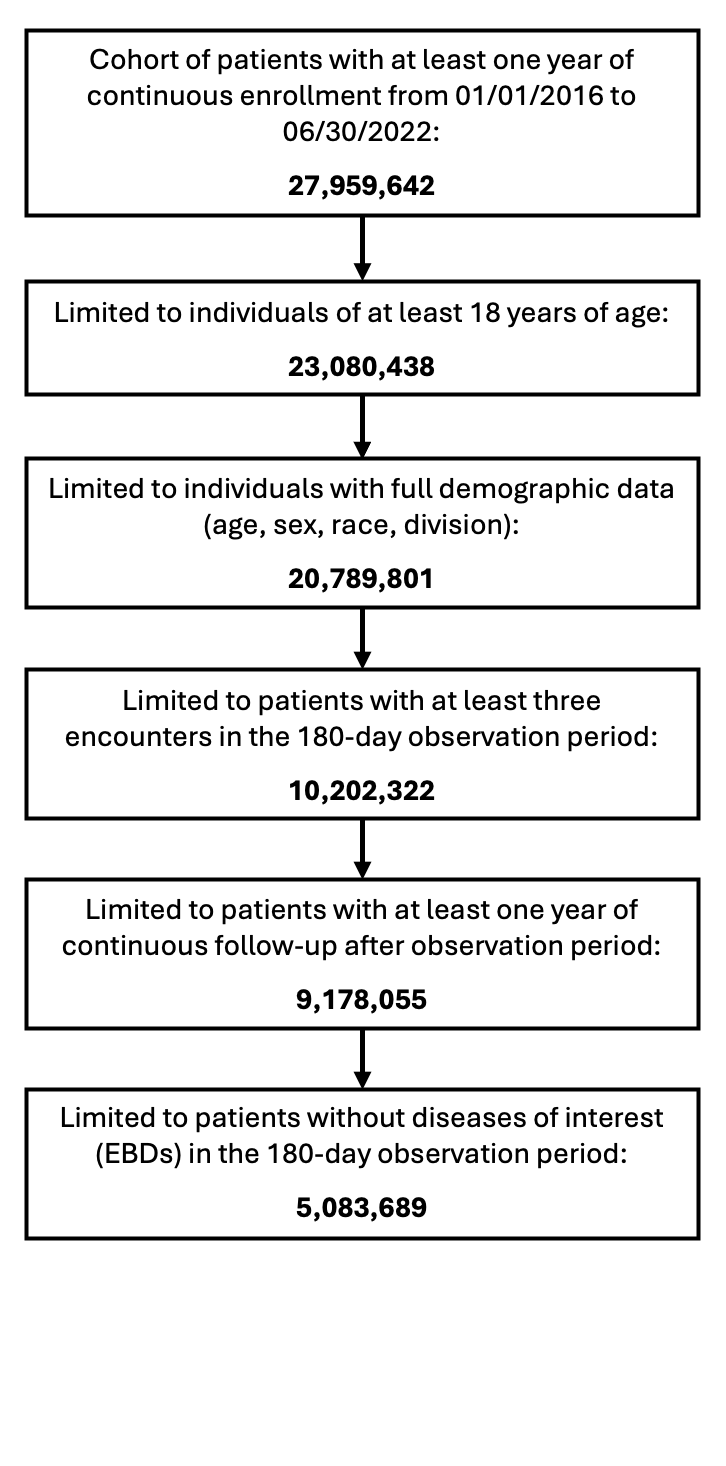
**

**Figure S1:** Schematic of analytical cohort construction showing the number of patients at each stage of cohort construction.

A cohort was constructed consisting of patients with at least one SD diagnosis (ICD-10 codes: L21.0, L21.8, L21.9) and at least one year of continuous enrollment from 01/01/2016 to 06/30/2022. This cohort was filtered to patients of at least 18 years of age at enrollment. Because only the year of birth for each patient is available, the date of birth for all patients was assigned to July 1 of the year of birth. Age at enrollment was calculated as the difference between this assigned birth date and the date of enrollment. Patients with any missing demographic information (age, sex, race, and division) were dropped from the cohort to ensure that all analyses could be performed with the same group of individuals. At the stage where patients with missing demographic information were dropped, 13 patients had a missing age, 2,482 had a missing sex, 1,966,849 had a missing race, and 460,193 had a missing division. Patients were then filtered based on the number of visits in the initial 180-day observation period, keeping only patients who had at least three visits on unique days, who represent approximately the top 50% of healthcare users. This was done to ensure consistent healthcare usage. Patients without an adequate follow-up period of one year after the initial 180-day observation period were removed. Finally, to identify patients with “new” diagnoses during the analysis period, we removed patients who had a diagnosis of any disease of interest within the initial 180-day observation period.

In secondary analyses, a diagnosis of type 2 diabetes was defined using one instance of ICD-10 codes E1100, E1101, E1110, E1111, E1121, E1122, E1129, E11311, E11319, E11321, E113211, E113212, E113213, E113219, E11329, E113291, E113292, E113293, E113299, E11331, E113311, E113312, E113313, E113319, E11339, E113391, E113392, E113393, E113399, E11341, E113411, E113412, E113413, E113419, E11349, E113491, E113492, E113493, E113499, E11351, E113511, E113512, E113513, E113519, E113521, E113522, E113523, E113529, E113531, E113532, E113533, E113539, E113541, E113542, E113543, E113549, E113551, E113552, E113553, E113559, E11359, E113591, E113592, E113593, E113599, E1136, E1137X1, E1137X2, E1137X3, E1137X9, E1139, E1140, E1141, E1142, E1143, E1144, E1149, E1151, E1152, E1159, E11610, E11618, E11620, E11621, E11622, E11628, E11630, E11638, E11641, E11649, E1165, E1169, E118, and E119.

A diagnosis of obesity was defined using one instance of ICD-10 codes E6601, E6609, E662, E668, and E669.

Smoking was defined using one instance of ICD-10 codes F17210, F17211, F17213, F17218, F17219, F17220, F17221, F17223, F17228, F17229, F17290, F17291, F17293, F17298, and F17299.

Alcohol use disorder was defined using one instance of ICD-10 codes F1010, F1011, F10120, F10121, F10129, F10130, F10131, F10132, F10139, F1014, F10150, F10151, F10159, F10180, F10181, F10182, F10188, F1019, F1020, F1021, F10220, F10221, F10229, F10230, F10231, F10232, F10239, F1024, F10250, F10251, F10259, F1026, F1027, F10280, F10281, F10282, F10288, F1029, F1090, F1091, F10920, F10921, F10929, F10930, F10931, F10932, F10939, F1094, F10950, F10951, F10959, F1096, F1097, F10980, F10981, F10982, F10988, and F1099.

An individual was considered exposed to comorbid type 2 diabetes, obesity, smoking, and alcohol use disorder if the corresponding ICD-10 code was logged prior to the date that the individual was censored from analysis.

**Supplemental Tables**

**Supplemental Table 1.** Table of diseases and corresponding ICD-10 codes.

| **Disease** | **ICD-10 codes** |
| --- | --- |
| **Skin** |  |
| Seborrheic dermatitis (SD) | L210,L218,L219 |
| Atopic dermatitis | L2081,L2082,L2083,L2084,L2089,L209 |
| Alopecia areata | L630,L631,L632,L638,L639 |
| Contact dermatitis | L230,L231,L232,L233,L234,L235,L236,L237,L2381,L2389,L239,L240,L241,L242,L243,L244,L245,L246,L247,L2481,L2489,L249,L24A0,L24A1,L24A2,L24A9,L24B0,L24B1,L24B2,L24B3,L250,L251,L252,L253,L254,L255,L258,L259 |
| Psoriasis | L400,L401,L402,L403,L404,L4050,L4051,L4052,L4053,L4054,L4059,L408,L409 |
| Rosacea | L710,L711,L718,L719 |
| Hidradenitis suppurativa | L732 |
| **Respiratory** |  |
| Asthma | J4520,J4521,J4522,J4530,J4531,J4532,J4540,J4541,J4542,J4550,J4551,J4552,J45901,J45902,J45909,J45990,J45991,J45998,J8283,J678 |
| Rhinosinusitis | J300,J301,J302,J305,J308,J3081,J3089,J309,J310,J320,J321,J322,J323,J324,J328,J329 |
| Chronic obstructive pulmonary disease (COPD) | J440,J441,J4481,J4489,J449 |
| Sarcoidosis | D860,D861,D862,D863,D8681,D8682,D8683,D8684,D8685,D8686,D8687,D8689,D869 |
| Pulmonary hypertension | I270,I272,I2720,I2721,I2722,I2723,I2724,I2729 |
| **Gastrointestinal** |  |
| Eosinophilic esophagitis | K200 |
| Gastroesophageal reflux disease (GERD) | K210,K2100,K2101,K219 |
| Food allergy | Z91010,Z91011,Z91012,Z91013,Z91014,Z91018,T7800XA,T7800XD,T7800XS,T7801XA,T7801XD,T7801XS,T7802XA,T7802XD,T7802XS,T7803XA,T7803XD,T7803XS,T7804XA,T7804XD,T7804XS,T7805XA,T7805XD,T7805XS,T7806XA,T7806XD,T7806XS,T7807XA,T7807XD,T7807XS,T7808XA,T7808XD,T7808XS,T7809XA,T7809XD,T7809XS,T781XXA,T781XXD,T781XXS,J305,L236,L246,L254,L272,K522,K5221,K5222,K5229 |
| Inflammatory bowel disease (IBD) | K5000,K50011,K50012,K50013,K50014,K50018,K50019,K5010,K50111,K50112,K50113,K50114,K50118,K50119,K5080,K50811,K50812,K50813,K50814,K50818,K50819,K5090,K50911,K50912,K50913,K50914,K50918,K50919,K5100,K51011,K51012,K51013,K51014,K51018,K51019,K5120,K51211,K51212,K51213,K51214,K51218,K51219,K5130,K51311,K51312,K51313,K51314,K51318,K51319,K5140,K51411,K51412,K51413,K51414,K51418,K51419,K5150,K51511,K51512,K51513,K51514,K51518,K51519,K5180,K51811,K51812,K51813,K51814,K51818,K51819,K5190,K51911,K51912,K51913,K51914,K51918,K51919 |
| Celiac disease | K900,L130 |
| Diverticulosis | K5700,K5701,K5710,K5711,K5712,K5713,K5720,K5721,K5730,K5731,K5732,K5733,K5740,K5741,K5750,K5751,K5752,K5753,K5780,K5781,K5790,K5791,K5792,K5793 |
| **Ocular** |  |
| Ocular allergy | H1010,H1011,H1012,H1013 |
| Macular degeneration | H3531,H353110,H353111,H353112,H353113,H353114,H353120,H353121,H353122,H353123,H353124,H353130,H353131,H353132,H353133,H353134,H353190,H353191,H353192,H353193,H353194,H3532,H353210,H353211,H353212,H353213,H353220,H353221,H353222,H353223,H353230,H353231,H353232,H353233,H353290,H353291,H353292,H353293,H35361,H35362,H35363,H35369 |
| Dry eye | H04121,H04122,H04123,H04129,H02881,H02882,H02883,H02884,H02885,H02886,H02889,H0288A,H0288B,H01001,H01002,H01003,H01004,H01005,H01006,H01009,H0100A,H0100B,H01011,H01012,H01013,H01014,H01015,H01016,H01019,H0101A,H0101B,H01021,H01022,H01023,H01024,H01025,H01026,H01029,H0102A,H0102B,H10509,H16223 |
| Glaucoma | H40001,H40002,H40003,H40009,H40011,H40012,H40013,H40019,H40021,H40022,H40023,H40029,H40031,H40032,H40033,H40039,H40041,H40042,H40043,H40049,H40051,H40052,H40053,H40059,H40061,H40062,H40063,H40069,H4010X0,H4010X1,H4010X2,H4010X3,H4010X4,H401110,H401111,H401112,H401113,H401114,H401120,H401121,H401122,H401123,H401124,H401130,H401131,H401132,H401133,H401134,H401190,H401191,H401192,H401193,H401194,H4011X0,H4011X1,H4011X2,H4011X3,H4011X4,H401210,H401211,H401212,H401213,H401214,H401220,H401221,H401222,H401223,H401224,H401230,H401231,H401232,H401233,H401234,H401290,H401291,H401292,H401293,H401294,H401310,H401311,H401312,H401313,H401314,H401320,H401321,H401322,H401323,H401324,H401330,H401331,H401332,H401333,H401334,H401390,H401391,H401392,H401393,H401394,H401410,H401411,H401412,H401413,H401414,H401420,H401421,H401422,H401423,H401424,H401430,H401431,H401432,H401433,H401434,H401490,H401491,H401492,H401493,H401494,H40151,H40152,H40153,H40159,H4020X0,H4020X1,H4020X2,H4020X3,H4020X4,H40211,H40212,H40213,H40219,H402210,H402211,H402212,H402213,H402214,H402220,H402221,H402222,H402223,H402224,H402230,H402231,H402232,H402233,H402234,H402290,H402291,H402292,H402293,H402294,H40231,H40232,H40233,H40239,H40241,H40242,H40243,H40249,H4030X0,H4030X1,H4030X2,H4030X3,H4030X4,H4031X0,H4031X1,H4031X2,H4031X3,H4031X4,H4032X0,H4032X1,H4032X2,H4032X3,H4032X4,H4033X0,H4033X1,H4033X2,H4033X3,H4033X4,H4040X0,H4040X1,H4040X2,H4040X3,H4040X4,H4041X0,H4041X1,H4041X2,H4041X3,H4041X4,H4042X0,H4042X1,H4042X2,H4042X3,H4042X4,H4043X0,H4043X1,H4043X2,H4043X3,H4043X4,H4050X0,H4050X1,H4050X2,H4050X3,H4050X4,H4051X0,H4051X1,H4051X2,H4051X3,H4051X4,H4052X0,H4052X1,H4052X2,H4052X3,H4052X4,H4053X0,H4053X1,H4053X2,H4053X3,H4053X4,H4060X0,H4060X1,H4060X2,H4060X3,H4060X4,H4061X0,H4061X1,H4061X2,H4061X3,H4061X4,H4062X0,H4062X1,H4062X2,H4062X3,H4062X4,H4063X0,H4063X1,H4063X2,H4063X3,H4063X4,H40811,H40812,H40813,H40819,H40821,H40822,H40823,H40829,H40831,H40832,H40833,H40839,H4089,H409,H42,H44511,H44512,H44513,H44519 |
| Uveitis | D8683,H209,H20041,H20042,H20043,H20049,H2010,H2011,H2012,H2013,H20021,H20022,H20023,H20029,H2000,H20011,H20012,H20013,H20019,H3020,H3021,H3022,H3023,H35061,H35062,H35063,H35069,H20821,H20822,H20823,H20829,H30811,H30812,H30813,H30819,H44111,H44112,H44113,H44119,H44131,H441312,H441313,H441319,H30011,H30012,H30013,H30019,H30021,H30022,H30023,H30029,H30031,H30032,H30033,H30039,H30041,H30042,H30043,H30049,H30141,H30142,H30143,H30149,H30891,H30892,H30893,H30899,H3090,H3091,H3092,H3093,H3122,H318,B0232,B0051,A5143,A5432,H32,B5800,B5801,B5809,H20031,H20032,H20033,H20039,H44001,H44002,H44003,H44009,H4419,H44121,H44122,H44123,H44129,H30101,H30102,H30103,H30109,H30111,H30112,H30113,H30119,H30121,H30122,H30123,H30129,H30131,H30132,H30133,H30139,B259,A1853,H20051,H20052,H20053,H20059,H20811,H20812,H20813,H20819 |

**Supplemental Table 2.** Cohort characteristics when defining SD diagnosis using at least two instances of SD-related ICD-10 codes.

|  | **Full cohort (as in Table 1)**  **N=5,083,689** | **≥ 2 SD Diagnoses**  **N=58,791** | **< 2 SD Diagnoses**  **N=5,024,898** |
| --- | --- | --- | --- |
| **Age at enrollment (years), median (IQR)** | 57.54 (40.53, 67.64) | 64.96 (46.42, 70.55) | 57.54 (40.53, 67.55) |
| **Sex, count (% total)** |  |  |  |
| Female | 3,000,186 (59.02%) | 29,855 (50.78%) | 2,970,331 (59.11%) |
| Male | 2,083,503 (40.98%) | 28,936 (49.22%) | 2,054,567 (40.89%) |
| **Race, count (% total)** |  |  |  |
| White | 3,733,651 (73.44%) | 46,867 (79.72%) | 3,686,784 (73.37%) |
| Non-White | 1,350,038 (26.56%) | 11,924 (20.28%) | 1,338,114 (26.63%) |
| **Division at time of enrollment, count (% total)** |  |  |  |
| East North Central (IL, IN, MI, OH, WI) | 735,732 (14.47%) | 7,258 (12.35%) | 728,474 (14.50%) |
| East South Central (AL, KY, MS, TN) | 222,478 (4.38%) | 2,316 (3.94%) | 220,162 (4.38%) |
| Middle Atlantic (NJ, NY, PA) | 417,151 (8.21%) | 5,304 (9.02%) | 411,847 (8.20%) |
| Mountain (AZ, CO, ID, MT, NV, NM, UT, WY) | 486,606 (9.57%) | 5,021 (8.54%) | 481,585 (9.58%) |
| New England (CT, ME, MA, NH, RI, VT) | 209,672 (4.12%) | 2,324 (3.95%) | 207,348 (4.13%) |
| Pacific (AK, CA, HI, OR, WA) | 606,121 (11.92%) | 7,360 (12.52%) | 598,761 (11.92%) |
| South Atlantic (DE, DC, FL, GA, MD, NC, SC, VA, WV) | 1,205,337 (23.71%) | 18,238 (31.02%) | 1,187,099 (23.62%) |
| West North Central (IA, KS, MN, MO, NE, ND, SD) | 476,609 (9.38%) | 4,120 (7.01%) | 472,489 (9.40%) |
| West South Central (AR, LA, OK, TX) | 723,983 (14.24%) | 6,850 (11.65%) | 717,133 (14.27%) |

**Supplemental Table 3.** Cohort characteristics after removing AD and psoriasis patients.

|  | **Full cohort**  **N=4,871,691** | **SD Diagnosis**  **N=153,949** | **No SD Diagnosis**  **N=4,717,742** |
| --- | --- | --- | --- |
| **Age at enrollment (years), median (IQR)** | 57.54 (40.53, 67.55) | 64.63 (44.53, 70.55) | 57.38 (40.43, 67.55) |
| **Sex, count (% total)** |  |  |  |
| Female | 2,870,002 (58.91%) | 81,608 (53.01%) | 2,788,394 (59.10%) |
| Male | 2,001,689 (41.09%) | 72,341 (46.99%) | 1,929,348 (40.90%) |
| **Race, count (% total)** |  |  |  |
| White | 3,572,182 (73.33%) | 124,200 (80.68%) | 3,447,982 (73.09%) |
| Non-White | 1,299,509 (26.67%) | 29,749 (19.32%) | 1,269,760 (26.91%) |
| **Division at time of enrollment, count (% total)** |  |  |  |
| East North Central (IL, IN, MI, OH, WI) | 708,555 (14.54%) | 19,109 (12.41%) | 689,446 (14.61%) |
| East South Central (AL, KY, MS, TN) | 214,436 (4.40%) | 6,297 (4.09%) | 208,139 (4.41%) |
| Middle Atlantic (NJ, NY, PA) | 395,806 (8.12%) | 12,870 (8.36%) | 382,936 (8.12%) |
| Mountain (AZ, CO, ID, MT, NV, NM, UT, WY) | 466,363 (9.57%) | 13,842 (8.99%) | 452,521 (9.59%) |
| New England (CT, ME, MA, NH, RI, VT) | 199,689 (4.10%) | 5,928 (3.85%) | 193,761 (4.11%) |
| Pacific (AK, CA, HI, OR, WA) | 580,542 (11.92%) | 19,745 (12.83%) | 560,797 (11.89%) |
| South Atlantic (DE, DC, FL, GA, MD, NC, SC, VA, WV) | 1,149,714 (23.60%) | 45,859 (29.79%) | 1,103,855 (23.40%) |
| West North Central (IA, KS, MN, MO, NE, ND, SD) | 461,098 (9.46%) | 11,619 (7.55%) | 449,479 (9.53%) |
| West South Central (AR, LA, OK, TX) | 695,488 (14.28%) | 18,680 (12.13%) | 676,808 (14.35%) |

**Supplemental Table 4.** Cohort characteristics for an expanded cohort containing patients with at least one encounter during the initial 180-day observation period.

|  | **Full cohort**  **N=8,742,803** | **SD Diagnosis**  **N=268,911** | **No SD Diagnosis**  **N=8,473,892** |
| --- | --- | --- | --- |
| **Age at enrollment (years), median (IQR)** | 52.70 (36.53, 66.13) | 59.04 (39.87, 68.55) | 52.54 (36.53, 65.96) |
| **Sex, count (% total)** |  |  |  |
| Female | 4,884,761 (55.87%) | 140,981 (52.43%) | 4,743,780 (55.98%) |
| Male | 3,858,042 (44.13%) | 127,930 (47.57%) | 3,730,112 (44.02%) |
| **Race, count (% total)** |  |  |  |
| White | 6,375,291 (72.92%) | 213,455 (79.38%) | 6,161,836 (72.72%) |
| Non-White | 2,367,512 (27.08%) | 55,456 (20.62%) | 2,312,056 (27.28%) |
| **Division at time of enrollment, count (% total)** |  |  |  |
| East North Central (IL, IN, MI, OH, WI) | 1,316,615 (15.06%) | 33,601 (12.50%) | 1,283,014 (15.14%) |
| East South Central (AL, KY, MS, TN) | 387,080 (4.43%) | 10,580 (3.93%) | 376,500 (4.44%) |
| Middle Atlantic (NJ, NY, PA) | 680,765 (7.79%) | 22,781 (8.47%) | 657,984 (7.76%) |
| Mountain (AZ, CO, ID, MT, NV, NM, UT, WY) | 847,252 (9.69%) | 24,152 (8.98%) | 823,100 (9.71%) |
| New England (CT, ME, MA, NH, RI, VT) | 342,710 (3.92%) | 10,441 (3.88%) | 332,269 (3.92%) |
| Pacific (AK, CA, HI, OR, WA) | 1,003,932 (11.48%) | 33,843 (12.59%) | 970,089 (11.45%) |
| South Atlantic (DE, DC, FL, GA, MD, NC, SC, VA, WV) | 2,011,212 (23.00%) | 78,387 (29.15%) | 1,932,825 (22.81%) |
| West North Central (IA, KS, MN, MO, NE, ND, SD) | 881,849 (10.09%) | 20,961 (7.79%) | 860,888 (10.16%) |
| West South Central (AR, LA, OK, TX) | 1,271,388 (14.54%) | 34,165 (12.70%) | 1,237,223 (14.60%) |

**Supplemental Table 5.** The hazard ratio (HR) and 95% confidence interval (CI) of SD diagnosis after EBD diagnosis using a multivariable Cox proportional hazards model for the following secondary analyses: adjusting for AD and psoriasis, removing AD and psoriasis patients, defining SD with at least two instances of diagnosis codes instead of one, and utilizing an expanded cohort of patients with at least one encounter during the 180-day observation period.

| **Disease** | **Adjust for AD & psoriasis: HR (95% CI)^a^** | **Remove AD & psoriasis: HR (95% CI)^b^** | **Define SD and EBDs with two diagnosis codes: HR (95% CI)^b^** | **Expanded cohort with ≥ 1 encounter in the observation period: HR (95% CI)^b^** |
| --- | --- | --- | --- | --- |
| **Skin** |  |  |  |  |
| Atopic dermatitis | N/A | N/A | 3.85 (3.60, 4.11) | 2.60 (2.54, 2.66) |
| Alopecia areata | 3.03 (2.83, 3.24) | 3.48 (3.23, 3.76) | 5.54 (4.80, 6.39) | 3.65 (3.46, 3.85) |
| Contact dermatitis | 1.64 (1.61, 1.67) | 1.85 (1.81, 1.89) | 2.92 (2.77, 3.07) | 1.97 (1.94, 2.01) |
| Psoriasis | N/A | N/A | 3.07 (2.89, 3.26) | 2.80 (2.73, 2.86) |
| Rosacea | 2.57 (2.51, 2.63) | 2.88 (2.81, 2.95) | 4.31 (4.11, 4.51) | 2.98 (2.92, 3.04) |
| Hidradenitis suppurativa | 1.63 (1.48, 1.79) | 1.71 (1.54, 1.91) | 2.22 (1.76, 2.79) | 1.81 (1.68, 1.96) |
| **Respiratory** |  |  |  |  |
| Asthma | 1.12 (1.10, 1.14) | 1.15 (1.13, 1.18) | 1.20 (1.15, 1.26) | 1.19 (1.17, 1.21) |
| Rhinosinusitis | 1.28 (1.26, 1.29) | 1.32 (1.30, 1.34) | 1.62 (1.57, 1.66) | 1.38 (1.36, 1.39) |
| COPD | 0.90 (0.88, 0.92) | 0.91 (0.89, 0.94) | 0.96 (0.92, 1.00) | 0.94 (0.92, 0.96) |
| Sarcoidosis | 1.19 (1.06, 1.35) | 1.29 (1.13, 1.47) | 1.32 (1.02, 1.71) | 1.21 (1.08, 1.35) |
| Pulmonary hypertension | 0.96 (0.93, 1.00) | 0.97 (0.93, 1.01) | 0.93 (0.86, 1.01) | 1.00 (0.97, 1.03) |
| **Gastrointestinal** |  |  |  |  |
| Eosinophilic esophagitis | 1.22 (1.09, 1.37) | 1.19 (1.04, 1.36) | 1.22 (0.90, 1.67) | 1.34 (1.22, 1.47) |
| GERD | 1.20 (1.18, 1.21) | 1.22 (1.21, 1.24) | 1.35 (1.32, 1.39) | 1.28 (1.27, 1.29) |
| Food allergy | 1.34 (1.29, 1.39) | 1.43 (1.37, 1.50) | 1.69 (1.53, 1.88) | 1.52 (1.47, 1.57) |
| IBD | 1.17 (1.12, 1.23) | 1.22 (1.16, 1.29) | 1.24 (1.10, 1.41) | 1.28 (1.23, 1.34) |
| Celiac disease | 1.41 (1.30, 1.53) | 1.44 (1.32, 1.59) | 1.56 (1.28, 1.90) | 1.59 (1.49, 1.71) |
| Diverticulosis | 1.18 (1.16, 1.20) | 1.21 (1.19, 1.23) | 1.33 (1.29, 1.38) | 1.24 (1.23, 1.26) |
| **Ocular** |  |  |  |  |
| Ocular allergy | 1.45 (1.39, 1.51) | 1.51 (1.44, 1.58) | 1.93 (1.69, 2.19) | 1.59 (1.53, 1.64) |
| Macular degeneration | 1.26 (1.23, 1.28) | 1.29 (1.26, 1.32) | 1.39 (1.33, 1.46) | 1.29 (1.27, 1.32) |
| Dry eye | 1.50 (1.48, 1.52) | 1.54 (1.51, 1.56) | 1.86 (1.81, 1.92) | 1.60 (1.58, 1.62) |
| Glaucoma | 1.15 (1.13, 1.17) | 1.17 (1.14, 1.19) | 1.32 (1.27, 1.37) | 1.19 (1.17, 1.21) |
| Uveitis | 1.20 (1.13, 1.27) | 1.22 (1.14, 1.30) | 1.34 (1.16, 1.53) | 1.24 (1.17, 1.30) |

^a^Analysis adjusted for age at enrollment, sex, race, division, diagnosis of AD, and diagnosis of psoriasis.

^b^Analysis adjusted for age at enrollment, sex, race, and division.

**Supplemental Table 6.** The hazard ratio (HR) and 95% confidence interval (CI) of EBD diagnosis after SD diagnosis using a multivariable Cox proportional hazards model for the following secondary analyses: adjusting for AD and psoriasis, removing AD and psoriasis patients, defining SD with at least two instances of diagnosis codes instead of one, and utilizing an expanded cohort of patients with at least one encounter during the 180-day observation period.

| **Disease** | **Adjust for AD & psoriasis: HR (95% CI)^a^** | **Remove AD & psoriasis: HR (95% CI)^b^** | **Define SD and EBDs with two diagnosis codes: HR (95% CI)^b^** | **Expanded cohort with ≥ 1 encounter in the observation period: HR (95% CI)^b^** |
| --- | --- | --- | --- | --- |
| **Skin** |  |  |  |  |
| Atopic dermatitis | N/A | N/A | 4.10 (3.87, 4.33) | 2.87 (2.81, 2.93) |
| Alopecia areata | 2.44 (2.26, 2.63) | 2.77 (2.55, 3.02) | 3.64 (3.10, 4.27) | 2.96 (2.78, 3.15) |
| Contact dermatitis | 1.76 (1.72, 1.79) | 1.97 (1.92, 2.01) | 3.08 (2.94, 3.23) | 2.09 (2.06, 2.13) |
| Psoriasis | N/A | N/A | 4.66 (4.43, 4.91) | 3.78 (3.69, 3.87) |
| Rosacea | 2.55 (2.49, 2.61) | 2.89 (2.81, 2.96) | 4.53 (4.32, 4.75) | 3.02 (2.96, 3.08) |
| Hidradenitis suppurativa | 1.35 (1.21, 1.49) | 1.41 (1.25, 1.59) | 2.36 (1.91, 2.91) | 1.52 (1.39, 1.65) |
| **Respiratory** |  |  |  |  |
| Asthma | 1.13 (1.11, 1.16) | 1.16 (1.13, 1.19) | 1.23 (1.17, 1.29) | 1.19 (1.17, 1.22) |
| Rhinosinusitis | 1.28 (1.27, 1.30) | 1.32 (1.30, 1.34) | 1.46 (1.42, 1.50) | 1.37 (1.35, 1.38) |
| COPD | 0.92 (0.89, 0.94) | 0.92 (0.90, 0.95) | 0.88 (0.84, 0.93) | 0.93 (0.91, 0.95) |
| Sarcoidosis | 1.28 (1.12, 1.46) | 1.30 (1.11, 1.51) | 1.36 (1.04, 1.79) | 1.33 (1.18, 1.50) |
| Pulmonary hypertension | 0.95 (0.92, 0.98) | 0.95 (0.92, 0.98) | 0.91 (0.85, 0.97) | 0.97 (0.95, 1.00) |
| **Gastrointestinal** |  |  |  |  |
| Eosinophilic esophagitis | 1.31 (1.17, 1.47) | 1.36 (1.20, 1.54) | 1.37 (1.03, 1.81) | 1.33 (1.21, 1.46) |
| GERD | 1.16 (1.14, 1.17) | 1.18 (1.16, 1.20) | 1.12 (1.09, 1.14) | 1.21 (1.20, 1.23) |
| Food allergy | 1.34 (1.29, 1.40) | 1.39 (1.33, 1.46) | 1.68 (1.53, 1.85) | 1.57 (1.52, 1.63) |
| IBD | 1.17 (1.11, 1.23) | 1.18 (1.11, 1.25) | 1.26 (1.11, 1.43) | 1.25 (1.19, 1.31) |
| Celiac disease | 1.31 (1.19, 1.44) | 1.37 (1.23, 1.53) | 1.58 (1.28, 1.95) | 1.58 (1.46, 1.71) |
| Diverticulosis | 1.15 (1.13, 1.17) | 1.17 (1.15, 1.19) | 1.24 (1.20, 1.29) | 1.19 (1.18, 1.21) |
| **Ocular** |  |  |  |  |
| Ocular allergy | 1.43 (1.37, 1.49) | 1.51 (1.44, 1.58) | 2.28 (2.03, 2.56) | 1.60 (1.54, 1.65) |
| Macular degeneration | 1.19 (1.16, 1.22) | 1.21 (1.18, 1.25) | 1.29 (1.23, 1.35) | 1.23 (1.20, 1.26) |
| Dry eye | 1.47 (1.45, 1.49) | 1.51 (1.48, 1.53) | 1.72 (1.67, 1.78) | 1.57 (1.55, 1.59) |
| Glaucoma | 1.11 (1.09, 1.13) | 1.13 (1.10, 1.15) | 1.17 (1.12, 1.22) | 1.15 (1.13, 1.18) |
| Uveitis | 1.16 (1.09, 1.23) | 1.21 (1.13, 1.29) | 1.46 (1.30, 1.64) | 1.20 (1.14, 1.27) |

^a^Analysis adjusted for age at enrollment, sex, race, division, diagnosis of AD, and diagnosis of psoriasis.

^b^Analysis adjusted for age at enrollment, sex, race, and division.

**Supplemental Table 7.** The hazard ratio (HR) and 95% confidence interval (CI) of SD diagnosis after EBD diagnosis using a multivariable Cox proportional hazards model for the following secondary analyses: adjusting for comorbid obesity & type 2 diabetes and additionally adjusting for environmental exposures to smoking and alcohol.

| **Disease** | **Adjust for type 2 diabetes and obesity: HR (95% CI)^a^** | **Adjust for type 2 diabetes, obesity, smoking, and alcohol: HR (95% CI)^b^** |
| --- | --- | --- |
| **Skin** |  |  |
| Atopic dermatitis | 2.44 (2.38, 2.51) | 2.43 (2.37, 2.49) |
| Alopecia areata | 3.39 (3.17, 3.63) | 3.35 (3.14, 3.59) |
| Contact dermatitis | 1.90 (1.86, 1.93) | 1.88 (1.84, 1.92) |
| Psoriasis | 2.71 (2.63, 2.78) | 2.75 (2.68, 2.83) |
| Rosacea | 2.78 (2.72, 2.84) | 2.72 (2.66, 2.78) |
| Hidradenitis suppurativa | 2.04 (1.86, 2.24) | 2.23 (2.03, 2.45) |
| **Respiratory** |  |  |
| Asthma | 1.25 (1.23, 1.28) | 1.28 (1.25, 1.30) |
| Rhinosinusitis | 1.36 (1.34, 1.38) | 1.36 (1.34, 1.38) |
| COPD | 0.98 (0.95, 1.00) | 1.17 (1.14, 1.19) |
| Sarcoidosis | 1.34 (1.19, 1.52) | 1.34 (1.18, 1.51) |
| Pulmonary hypertension | 1.05 (1.02, 1.09) | 1.07 (1.03, 1.11) |
| **Gastrointestinal** |  |  |
| Eosinophilic esophagitis | 1.25 (1.12, 1.41) | 1.23 (1.10, 1.38) |
| GERD | 1.30 (1.29, 1.32) | 1.33 (1.32, 1.35) |
| Food allergy | 1.52 (1.46, 1.58) | 1.53 (1.47, 1.59) |
| IBD | 1.24 (1.18, 1.31) | 1.27 (1.21, 1.33) |
| Celiac disease | 1.53 (1.41, 1.65) | 1.52 (1.40, 1.65) |
| Diverticulosis | 1.25 (1.23, 1.27) | 1.27 (1.25, 1.29) |
| **Ocular** |  |  |
| Ocular allergy | 1.56 (1.50, 1.62) | 1.54 (1.48, 1.61) |
| Macular degeneration | 1.25 (1.22, 1.28) | 1.24 (1.21, 1.27) |
| Dry eye | 1.53 (1.51, 1.55) | 1.51 (1.49, 1.53) |
| Glaucoma | 1.19 (1.17, 1.22) | 1.18 (1.16, 1.20) |
| Uveitis | 1.26 (1.19, 1.33) | 1.26 (1.19, 1.34) |

^a^Analysis adjusted for age at enrollment, sex, race, division, diagnosis of type 2 diabetes, and diagnosis of obesity.

^b^Analysis adjusted for age at enrollment, sex, race, division, diagnosis of type 2 diabetes, diagnosis of obesity, smoking, and alcohol use.

**Supplemental Table 8.** The hazard ratio (HR) and 95% confidence interval (CI) of EBD diagnosis after SD diagnosis using a multivariable Cox proportional hazards model for the following secondary analyses: adjusting for comorbid obesity & type 2 diabetes and additionally adjusting for environmental exposures to smoking and alcohol.

| **Disease** | **Adjust for type 2 diabetes and obesity: HR (95% CI)^a^** | **Adjust for type 2 diabetes, obesity, smoking, and alcohol: HR (95% CI)^b^** |
| --- | --- | --- |
| **Skin** |  |  |
| Atopic dermatitis | 2.69 (2.63, 2.76) | 2.66 (2.60, 2.72) |
| Alopecia areata | 2.77 (2.57, 2.99) | 2.74 (2.54, 2.95) |
| Contact dermatitis | 2.03 (1.99, 2.07) | 2.01 (1.97, 2.04) |
| Psoriasis | 3.51 (3.41, 3.60) | 3.49 (3.39, 3.58) |
| Rosacea | 2.81 (2.74, 2.87) | 2.75 (2.69, 2.82) |
| Hidradenitis suppurativa | 1.53 (1.38, 1.69) | 1.57 (1.41, 1.74) |
| **Respiratory** |  |  |
| Asthma | 1.19 (1.16, 1.21) | 1.18 (1.16, 1.21) |
| Rhinosinusitis | 1.33 (1.31, 1.35) | 1.32 (1.30, 1.34) |
| COPD | 0.94 (0.92, 0.96) | 0.98 (0.96, 1.00) |
| Sarcoidosis | 1.35 (1.18, 1.54) | 1.34 (1.17, 1.53) |
| Pulmonary hypertension | 0.96 (0.93, 0.99) | 0.96 (0.93, 0.99) |
| **Gastrointestinal** |  |  |
| Eosinophilic esophagitis | 1.36 (1.21, 1.52) | 1.34 (1.20, 1.50) |
| GERD | 1.18 (1.17, 1.20) | 1.19 (1.17, 1.20) |
| Food allergy | 1.48 (1.43, 1.55) | 1.48 (1.42, 1.54) |
| IBD | 1.20 (1.14, 1.27) | 1.20 (1.14, 1.27) |
| Celiac disease | 1.43 (1.30, 1.57) | 1.41 (1.29, 1.55) |
| Diverticulosis | 1.17 (1.15, 1.18) | 1.17 (1.15, 1.18) |
| **Ocular** |  |  |
| Ocular allergy | 1.53 (1.46, 1.59) | 1.51 (1.45, 1.57) |
| Macular degeneration | 1.21 (1.18, 1.24) | 1.21 (1.18, 1.23) |
| Dry eye | 1.51 (1.49, 1.53) | 1.49 (1.47, 1.51) |
| Glaucoma | 1.13 (1.11, 1.16) | 1.12 (1.10, 1.14) |
| Uveitis | 1.20 (1.13, 1.27) | 1.19 (1.12, 1.27) |

^a^Analysis adjusted for age at enrollment, sex, race, division, diagnosis of type 2 diabetes, and diagnosis of obesity.

^b^Analysis adjusted for age at enrollment, sex, race, division, diagnosis of type 2 diabetes, diagnosis of obesity, smoking, and alcohol use.
